# Three‐dimensional tissue engineering and organoid technologies for retinal regeneration and therapy

**DOI:** 10.1002/btm2.70051

**Published:** 2025-08-14

**Authors:** Yiqi Wang, Douglas Jiang, Qinglong Wang, Yun Cao, Hong Guo, Yi Lu, Feng Tian

**Affiliations:** ^1^ Department of Neurology, Beth Israel Deaconess Medical Center Harvard Medical School Boston Massachusetts USA; ^2^ Department of Biology Georgia Institute of Technology Atlanta Georgia USA; ^3^ School of Arts and Sciences Tufts University Medford Massachusetts USA; ^4^ Department of Neurosurgery, Brigham and Women's Hospital Harvard Medical School Boston Massachusetts USA

**Keywords:** AMD (age‐related macular degeneration), bioprinting, glaucoma, organoid, photoreceptor, retina, RPE (retinal pigment epithelium)

## Abstract

The human eye, a masterpiece of evolution, orchestrates the intricate process of vision. The retina is a tissue with a layered structure that plays a critical role in converting light signals into neural impulses interpretable by the brain. Various eye conditions such as glaucoma, retinitis pigmentosa, age‐related macular degeneration, and other retinopathies are characterized by damage or degeneration in the retina. Recent strides in organoid cultivation and advanced three‐dimensional (3D) bioengineering technologies offer promising avenues for potential therapeutic interventions. Compared to traditional two‐dimensional cell culture models, which are non‐natural and limited in accuracy, 3D models, including organoids, electrospinning constructs, microfabrication‐based scaffolds, and hydrogel systems, are more delicate, especially in recapitulating tissue architecture, offering spatial patterning, and enabling vascularization. Retinal organoids are 3D multicellular structures derived from stem cells that can mimic the retina's layered architecture and functionality. However, their inherent complexity, including the presence of multiple differentiated cell types, may not be necessary for all disease modeling applications. In contrast, engineered 3D technologies can be tailored to specific retinal diseases by incorporating only the most relevant cell types, matrix stiffness, and spatial arrangements, offering greater experimental control and reproducibility in targeted therapeutic testing. In the following paper, we will discuss organoid generation in detail. Besides retinal organoids, bioprinting is another promising avenue for regenerative medicines. We further review a suite of 3D fabrication strategies, including inkjet and laser‐assisted bioprinting, electrospun scaffolds, and hydrogel systems, and evaluate their current and potential applications in modeling retinal diseases and developing translational therapies. We will also delve into the contemporary advancements in retinal therapies, particularly emphasizing the roles and prospects of organoid and engineered 3D technologies.

**Translational Impact Statement**

In this review, we explore the groundbreaking potential of three‐dimensional (3D) technologies for retinal regeneration, an array of cutting‐edge advancements that promise to revolutionize the treatment of vision loss and blindness. By leveraging stem cells to create 3D models of the human retina, organoids not only offer unprecedented insights into retinal development and disease but also pave the way for innovative therapies and drug testing, bringing new hope to millions worldwide affected by retinal conditions. This work highlights the transformative impact of retinal organoids, bioprinting, and stem cell therapy in bridging basic research and clinical application, marking retinal organoid technology, a significant milestone in regenerative medicine and vision restoration.

**Abbreviations**
ATCC: American Type Culture Collection
BMP4: Bone Morphogenetic Protein 4
DAPT: N‐[N‐(3,5‐Difluorophenacetyl)‐L‐alanyl]‐S‐phenylglycine t‐butyl ester (a γ‐secretase inhibitor)
DMEM: Dulbecco's Modified Eagle Medium
ES: Embryonic Stem (cells)
gfCDM: Growth factor‐free Chemically Defined Medium
hPSCs: Human Pluripotent Stem Cells
hRPE: Human Retinal Pigment Epithelium (cells)
KSR: KnockOut Serum Replacement
mTeSR1: Maintenance TeSR1 Medium (a defined medium for maintaining hPSCs)
PEG: Polyethylene Glycol
PLGA: Poly(lactic‐co‐glycolic acid)
RGDS: Arginine‐Glycine‐Aspartic Acid‐Serine peptide
TGFβ: Transforming Growth Factor Beta
UV: Ultraviolet

## 1 INTRODUCTION OF RETINA AND RETINAL DISORDERS

The retina is a laminated, light‐sensitive neural tissue located at the posterior segment of the eye that initiates the visual process by converting photons into neural signals. These signals are then transmitted to the brain, which interprets them to create vision. The eye functions not only as a sensory organ but also as an integral component of the central nervous system (CNS), owing to its linkage to the brain via the optic nerve. The visual transmission process predominantly involves the photoreceptor and glial cells, along with a network of interneurons and retinal ganglion cells (RGCs), which together form a sophisticated synaptic circuitry for image preprocessing.^1^ Malfunction or degeneration of the retina can lead to various retinal pathologies such as age‐related macular degeneration (AMD), glaucoma, and retinitis pigmentosa (RP).

**FIGURE 1:**
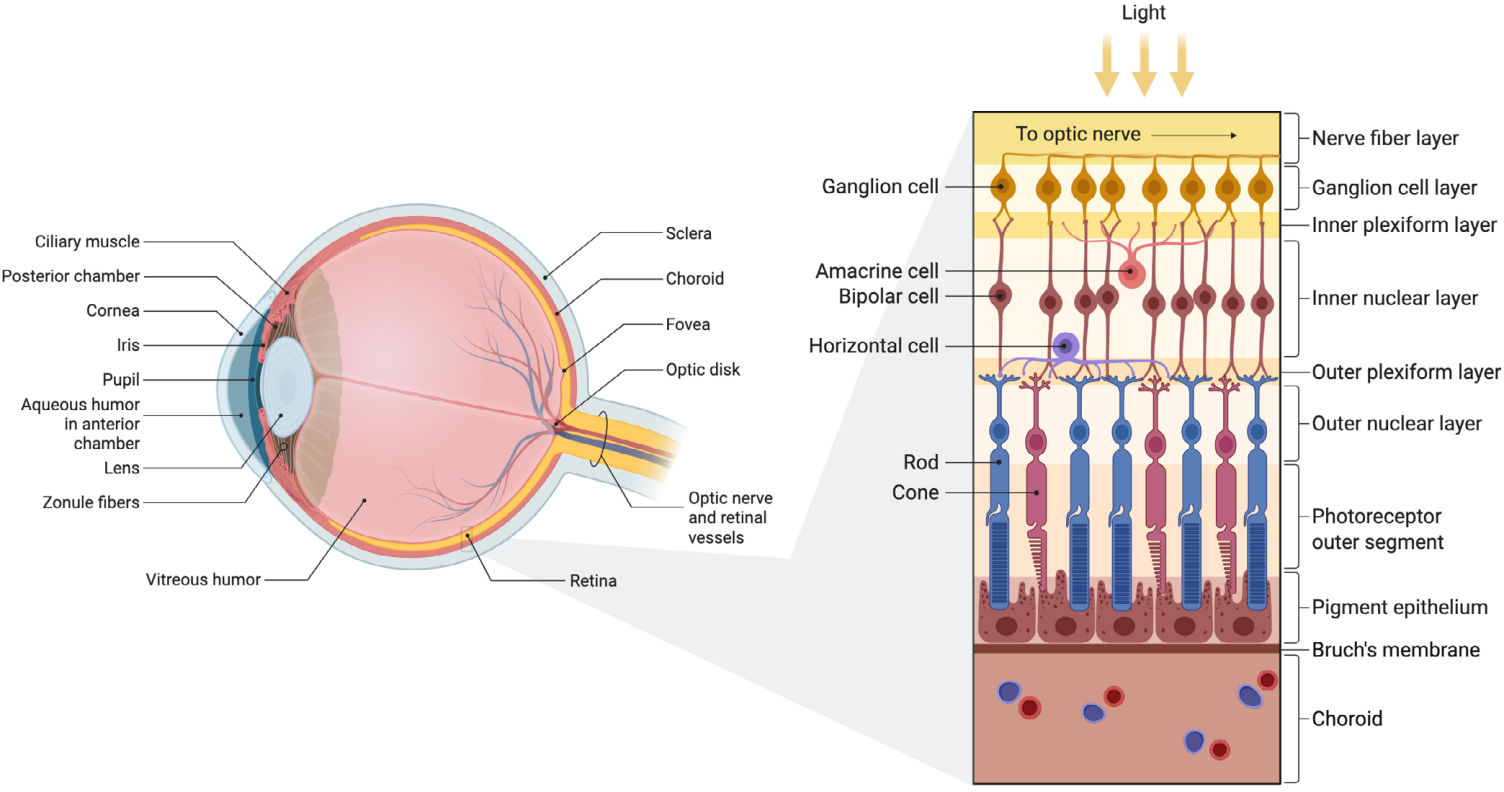
Anatomy of the eyeball and the structure of the retina. The retina is located at the back of the eye, and it can be subdivided into 10 different layers from the anterior to the posterior of the eyeball, including the inner limiting membrane, retinal nerve fiber layer, ganglion cell layer, inner plexiform layer, inner nuclear layer, outer plexiform layer, outer nuclear layer, external limiting membrane, photoreceptor layer, and retinal pigment epithelium.^2^ Created with BioRender.com

The retina can be subdivided into 10 different layers from the anterior to the posterior of the oculus, organized histologically from the vitreal surface to the choroidal side: (1) inner limiting membrane, (2) retinal nerve fiber layer, (3) ganglion cell layer, (4) inner plexiform layer, (5) inner nuclear layer, (6) outer plexiform layer, (7) outer nuclear layer, (8) external limiting membrane, (9) photoreceptor layer, and (10) retinal pigment epithelium (RPE) (Figure 1).^2^ These layers support a vertical and horizontal flow of visual information, involving synaptic connections across photoreceptors, bipolar cells, and ganglion cells, as well as lateral modulation by horizontal and amacrine cells.

Located near the back of the retinal sublayers, away from the pupil, are the photoreceptor cells, which are composed of rods and cones.^2^ Rod cells, which are highly sensitive to light, enable us to see in low‐light conditions, though they do not discern color. Conversely, cone cells, which operate in brighter lighting, facilitate color vision and provide sharp, detailed sight, allowing us to perceive fine details. While rods are more densely populated in the retina's periphery than in its center, cones are concentrated in the fovea, the central retina area crucial for sharp central vision. The human retina houses roughly 100–120 million rod cells.^3^ Moreover, there are three distinct types of cone cells, each responsive to varying light wavelengths: S (short)‐cones sensitive to blue light, M (medium)‐cones to green light, and L (long)‐cones to red light.^2^ Photoreceptors transmit data to interneurons, including horizontal, bipolar, and amacrine cells, which are responsible for processing light signals. These cells then connect with RGCs. The RGCs assimilate this data and convey it to the brain via their extended axons, forming the optic nerve.^4^ This layered organization allows the retina to perform significant preprocessing of visual information, such as contrast enhancement, motion detection, and edge detection before the signal even reaches the brain. The susceptibility of photoreceptors and RGCs to degeneration in many retinal diseases makes them key targets for emerging regenerative strategies, including stem cell transplantation and three‐dimensional (3D) tissue engineering.

### 1.1 Age‐related macular degeneration

**FIGURE 2:**
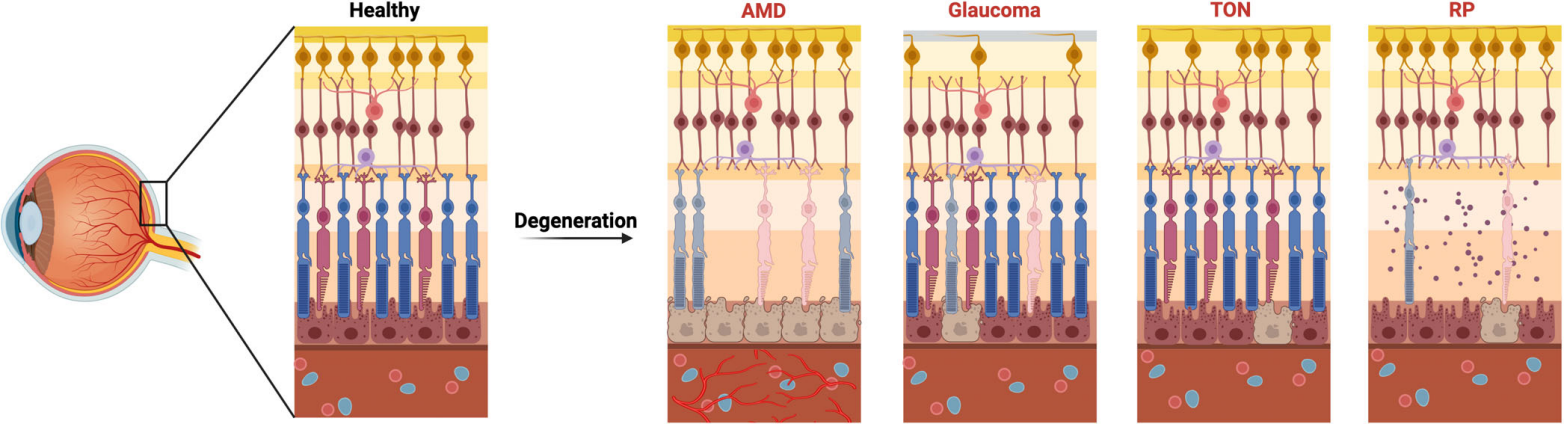
The comparison between healthy retina and retina with age‐related macular degeneration (AMD), glaucoma, traumatic optic neuropathy (TON), and retinitis pigmentosa (RP). This schematic illustrates the structural differences between a healthy retina and those impacted by AMD, glaucoma, TON, and RP. Created with BioRender.com

Compared to the healthy retina, AMD, the most prevalent and irreversible cause of blindness in the elderly population, is characterized by degeneration of retinal photoreceptors, RPE, and Bruch's membrane, along with potential atrophy of the choriocapillaris and disruption of the extracellular matrix^5^ (Figure 2). The classification of AMD is based on the presence of retinal atrophy and neovascularization, resulting in dry or wet forms, both leading to visual loss.^6^ AMD is frequently diagnosed in the advanced stages of disease progression when a portion of the visual field has already been compromised. The current treatment for wet AMD involves intravitreal injections of anti‐VEGF (vascular endothelial growth factor). Wet AMD occurs when abnormal blood vessels grow from the choroid into the subretinal space and damage the macula, leading to rapid vision loss. Intravitreal injections of anti‐VEGF agents are designed to inhibit the development of abnormal blood vessels in the eye. This intervention seeks to prevent vision loss and, in certain cases, can even enhance vision.^7^ However, while some patients experience temporary improvements in vision, one‐third fail to halt the deterioration of their eyesight. Meanwhile, dry AMD (also known as non‐neovascular or atrophic AMD) currently lacks any effective treatment options. Consequently, there is a pressing need for therapies aimed at restoring retinal function for individuals with AMD.^8^ Among 3D technologies, RPE replacement has become a promising strategy for treating AMD.^9^ Because the RPE supports photoreceptor health through phagocytosis of shed outer segments, nutrient transport, and retinoid cycling, its dysfunction plays a central role in both dry and wet AMD pathogenesis. Therefore, transplanting stem cell‐derived RPE monolayers or bioengineered constructs holds potential for both disease stabilization and vision restoration.

### 1.2 Glaucoma

Glaucoma, the second leading cause of blindness globally, often remains undetected until it is significantly advanced.^10^ It has been reported that only 10%–50% of glaucoma patients are aware of their condition.^11^ It primarily involves the progressive loss of RGCs and their axons, impairing visual transmission to the brain and resulting in blind spots^12^, ^13^ (Figure 2). Although glaucoma encompasses a group of heterogeneous disorders, elevated intraocular pressure (IOP) plays a critical role, even in patients with normal IOP levels.^14^ Currently, the only validated treatment to slow the degenerative progression of glaucoma involves lowering IOP, which can be achieved through medication to increase fluid outflow from the eye or reduce its production, as well as through surgical interventions like glaucoma filtration surgery. Studies on the key proteins associated with glaucoma have also provided new perspectives in finding potential therapeutic targets, such as myocilin (MYOC), latent transforming growth factor‐beta‐binding protein 2 (LTBP2), optineurin (OPTN), TANK‐binding kinase 1 (TBK1) and other genes implicated in inherited forms of glaucoma.^15^, ^16^ Also, reducing IOP by 20% can decrease the risk of disease progression by 50%.^17^ Eye drops and oral medications to lower IOP often require strict adherence to a daily regimen, which can be challenging for patients. Although selective laser trabeculoplasty (SLT) has been explored, its effectiveness remains limited.^18^ Surgical options like trabeculectomy or shunt implants can lower IOP but come with risks such as infection, bleeding, and vision loss. Furthermore, the irreversible damage to RGCs ultimately leads to blindness, with no effective treatment for this aspect of the disease. Because RGCs do not spontaneously regenerate and axonal connections to the brain are permanently lost, there is a critical need for neuroprotective and regenerative therapies that directly target RGC survival and repair. However, recent advancements in 3D technology, such as the development of retinal organoids, show promise. These organoids, which exhibit electrophysiological and metabolic functionalities, offer new possibilities. Specifically, injections of RGCs derived from retinal organoids into mice with optic neuropathy have shown improvements in visual function and have survived up to a month, highlighting a potential new frontier in glaucoma treatment that could 1 day extend to human therapy.^19^ Although full integration of transplanted RGCs remains a challenge, these 3D models provide valuable platforms for studying disease mechanisms, testing candidate drugs, and exploring cell‐based therapeutic strategies.

### 1.3 Traumatic optic neuropathy

Traumatic optic neuropathy (TON) refers to a direct or indirect insult that results in structural or functional injury to the axonal fiber within optic nerves^20^, ^21^, ^22^ (Figure 2). It is commonly associated with head trauma involving rapid acceleration and deceleration forces that can shear, stretch, or compress optic nerve axons. In severe cases, primary axotomy may occur, where axons are torn and disconnected from the cell body, leading to the formation of retraction bulbs and subsequent Wallerian degeneration.^23^ Traumatic axonal injury results in the degeneration of a population of axons distal and proximal to the injury, along with retrograde death of a subpopulation of RGCs. RGC soma and dendritic loss can precede axonal loss and optic nerve atrophy in both animal models and humans, regardless of whether there was a single severe impact or repetitive mild impacts.^24^, ^25^ This results in irreversible visual impairment due to the disruption of synaptic input to RGCs, the degeneration of their axons, and the permanent loss of connectivity between the retina and central visual targets.^26^ Complete primary axotomy, such as optic nerve crush, is a rare form of damage that occurs in cases of massive, widespread axonal damage.^27^ Although inertial forces do not cause complete primary axotomy, they can be strong enough to cause partial damage to the axon, as well as release damage‐associated molecular patterns resulting in secondary axon injury.^22^, ^28^ Secondary damage occurs hours to days after the initial insult because of neuroinflammation, oxidative stress, and excitotoxicity.^29^ Molecules expressed by stressed neurons directly trigger chronic microglia activation, which can persist for months or years in association with regions of axon degeneration and demyelination.^23^, ^30^ The treatment of TON varies but typically involves high‐dose corticosteroids to reduce inflammation or surgical interventions such as optic canal decompression to relieve pressure on the optic nerve. The effectiveness of high‐dose corticosteroids in treating TON remains controversial. Some studies suggest potential benefits, while others indicate no significant improvement in visual outcomes compared to no treatment.^31^ Surgical decompression of the optic canal aims to relieve pressure on the optic nerve, but the procedure carries significant risks, including infection, cerebrospinal fluid leaks, and damage to surrounding structures.^32^ Some cases may be managed by close observation to monitor any potential spontaneous recovery. Each approach is tailored based on the severity and specifics of the injury. Emerging technologies, such as retinal organoids and 3D bioprinting approaches, provide new platforms to study RGC loss and axonal regeneration in TON. Although functional axon reconnection to central targets remains a major barrier, these models offer opportunities for exploring neuroprotective compounds, stem cell‐based interventions, and scaffold‐assisted axonal guidance strategies.

### 1.4 Other retinal pathologies

In addition to the diseases mentioned above, other hereditary or complicating factors also contribute to retinal degeneration (Table 1; Figure 2). The retina serves as a vital source of visual information input due to its precise structure and sensitive functionality.^37^ Retinal degeneration inevitably leads to vision loss and a diminished quality of life. Currently, there is no effective therapy available for restoring retinal function. However, with the continuous advancement of organoid technology, retinal organoids have demonstrated light‐sensitive electrophysiological characteristics.^19^, ^38^ Here we present the contemporary advancements in retinal stem cell therapies, particularly emphasizing the roles and prospects of organoid and bioprinting technologies.

**TABLE 1:** Retinal disorders overview.

Disorder	Major features	Cause/mechanism	Current treatments
Age‐related macular degeneration (AMD)	Central vision loss; drusen; RPE and photoreceptor atrophy	Age‐related degeneration of macular RPE and photoreceptors; choroidal neovascularization in wet AMD^33^	Anti‐VEGF intravitreal injections (wet AMD); no effective treatment for dry AMD^34^; stem cell‐derived RPE transplantation under investigation
Retinitis pigmentosa (RP)	Night blindness; progressive peripheral vision loss; retinal bone‐spicule pigment deposits	Inherited gene mutations cause rod photoreceptor degeneration (followed by cone loss)^35^	No cure available; investigational photoreceptor or retinal progenitor cell transplantation in development^36^
Glaucoma	Gradual irreversible peripheral vision loss; optic disc cupping	Elevated intraocular pressure causes optic nerve (RGC) damage^11^	IOP‐lowering therapies (eye drops, laser, surgery) to slow progression; vision loss is irreversible^11^

Abbreviations: IOP, intraocular pressure; RGC, retinal ganglion cell; RPE, retinal pigment epithelium; VEGF, vascular endothelial growth factor.

## 2 STEM CELL THERAPIES IN RETINAL DEGENERATION

Stem cell therapy represents a branch of regenerative medicine that strives to restore or substitute impaired tissues or organs through the utilization of stem cells (Table 2). These stem cells are distinctive in their capability to transform into various cell types, ranging from muscle cells to neurons. Additionally, they hold the potential to self‐replicate, generating additional stem cells.

**TABLE 2:** Stem cell‐based therapies for retinal disorders.

Stem cell therapy	Cell source	Delivery method	Target disease	model	Key outcomes
RPE replacement	human embryonic stem cells (hESCs)/iPSCs	Subretinal injection (suspension or sheet)	AMD	Animal and human trials	Safe; some vision improvement^39^, ^40^
Photoreceptor transplant	iPSC‐derived precursors	Subretinal injection	RP, AMD	Mouse models	Partial vision restoration in animal models^41^
RPC transplantation	Fetal or pluripotent‐derived RPCs	Subretinal injection	RP and retinal degeneration	Preclinical models	Well‐tolerated; photoreceptor rescue^42^
RGC transplantation	hESCs/iPSCs (2D or organoid‐derived)	Intravitreal injection or scaffold delivery	Glaucoma, optic neuropathy	Animal models	Poor graft survival and integration^43^

Abbreviations: AMD, age‐related macular degeneration; hESCs, human embryonic stem cells; iPSCs, induced pluripotent stem cells; RGC, retinal ganglion cell; RP, retinitis pigmentosa; RPC, Retinal progenitor cells; RPE, retinal pigment epithelium; 2D, two‐dimensional.

Within the spectrum of stem cell therapies, three distinct cell types are commonly employed: embryonic stem cells (ESCs), somatic adult stem cells (ASCs), and induced pluripotent stem cells (iPSCs).^44^ ESCs are harvested from embryos and possess the remarkable ability to develop into any cell type within the human body. Somatic stem cells, in contrast, are located in various bodily tissues but have a more restricted capacity to differentiate when compared to ESCs. iPSCs represent adult cells that have undergone genetic reprogramming, reverting them to a state similar to ESCs.

### 2.1 Epithelium replacement

Among all the eye diseases discussed above, AMD stands as a primary contributor to visual impairment, with its pathogenic mechanisms originating from the RPE layer.^45^ RPE is a pigmented monolayer cell situated at the back of the eye between the retina and choroid.^46^ Since RPE does not need synaptic connections to obtain its mechanism, this makes RPE a great candidate for tissue transplantation. There are several approaches for RPE regeneration, including the pluripotent stem cell method. Together with novel strategies, RPE replacement becomes a promising strategy for treating AMD.^9^

RPE serves important functions in removing and degrading the tips of photoreceptor outer segments (POS). The degradation of POS distal tips in RPE is triggered by light through phagocytosis.^47^ Other than phagocytosis, retinoid recycling is another fundamental function in the visual cycle. Thus, the primary goal of constructing the RPE layer is to retain phagocytosis and retinoid recycling functions while maintaining its structure. It is noted that photoreceptors are interdigitated with microvilli on RPE to maintain a fixed plane status for the retina and produce nutrients and oxygen exchange from choriocapillaris to photoreceptors. This unique structure as well as pigmentation and melanogenesis constructs the full morphology of the RPE layer.^48^

ESCs and iPSCs are utilized in vitro to generate RPE. ESCs are recognized as pluripotent cells, possessing the capacity for indefinite proliferation and differentiation into any cell type, making them a potent option for regenerating RPEs.^44^ During the induction of spontaneous differentiation, a feeder layer of PA6 stromal cells is employed, and the fibroblast growth factor (bFGF) is withdrawn to induce the formation of pigmented clone structures.^49^ Another defined culture protocol required ESCs to be grown as embryoid bodies first, after which they were coated either with laminin and fibronectin or with gelatin until they formed visible pigmented colonies.^50^, ^51^

### 2.2 Photoreceptor transplants

Photoreceptors are specialized nerve cells found in the retina of the eye, responsible for converting light into electrical signals. These signals are then processed by other retinal neurons and sent to the brain via the optic nerve, resulting in the perception of vision. In the repair of the degenerate retina, it can be achieved by transplanting a photoreceptor.^52^

For AMD discussed above, we have proposed RPE transplantation, providing another treatment strategy that involves photoreceptor transplantation. Since the ultimate goal is to develop a patient‐specific autologous cell replacement, this technique's success in animal models with retinal degeneration would also provide tools to investigate how certain genetic mutations lead to disease.^53^ The objective was to assess the potential of patient‐specific photoreceptor precursor cells, derived from the neural retina layer of human iPSC‐derived eyecups, to mature into functional photoreceptors in animal models. Post‐mitotic precursor cells are identified as being the most effective candidates for retinal transplants.^41^, ^54^ Then, 150‐day‐old precursor cells were introduced into the subretinal region of young immunodeficient Rag1−/− × Crb1−/− mice, selecting this strain for their slower retinal deterioration and a higher likelihood of integrating the transplanted cells into their retinal architecture successfully.^52^ The rod photoreceptor replacement strategy in a mouse model of stationary night blindness included isolating cells from the mouse retina, which showed efficacy in photoreceptor cell replacement among different mouse models, providing a potential for photoreceptor replacement therapy.^52^ An alternative method for patient‐specific cell replacement that does not involve modifying donor cells genetically before transplantation is to utilize non‐matched stem (ES) cells or cells that are precursors to specific tissues. This method is undergoing testing in clinical trials for conditions like Stargardt macular dystrophy and AMD. In these trials, cells from the RPE derived from ES cells are injected beneath the retina of immunosuppressed patients.^55^ However, the use of cells from other individuals (allogeneic cells) has significant drawbacks. For example, in retinas damaged by hereditary diseases, the protective barrier between the blood and the retina may be compromised, allowing the patient's immune system to potentially attack the transplanted cells. This kind of transplant could necessitate ongoing immunomodulation treatment, which could pose additional risks to the patients.^56^ But in general, photoreceptor transplant therapy provides a promising treatment option, and how the cells grow and are structured can be further used in developing retinal organoids.

### 2.3 Retinal progenitor cells transplantation

Retinal progenitor cells (RPCs) are a subset of undifferentiated cells present during retinal development. These cells possess the remarkable ability to differentiate into all the diverse cell types present in the retina, establishing them as crucial contributors to the formation and progression of retinal tissues. RPC transplantation can also be a potential therapeutic option in repairing a degenerating retina. To differentiate human retinal progenitor cells (hRPCs), the cells were isolated from the fetal neural retina. The transplantation of hRPCs rescued the impairment of the retina, showing their promise in treating retina‐associated diseases. It is noted that the hRPC transplantation was injected subretinally and showed no adverse effects; it was well‐tolerated.^42^ Thus, hRPCs can be used in structuring 3D retina because of their high tolerance.

Currently, the effective therapeutic cells used in stem cell transplantation include RPCs, ESCs, iPSCs as a result of their proliferation and multidirectional differentiation potential.^57^ Promising studies on mesenchymal stem cell‐derived microvesicles (MVs) have also been carried out, due to their dynamic nature, as well as their regenerative nature in medicine.

### 2.4 RGCs transplantation

In addition to photoreceptor and RPE transplantation, investigators are exploring stem cell‐based replacement of RGCs to address optic neuropathies such as glaucoma. Glaucoma leads to vision loss through progressive RGC degeneration, and because these neurons do not spontaneously regenerate, transplantation of new RGCs has emerged as a potential restorative strategy.^43^, ^58^ Human pluripotent stem cells, including embryonic stem cells (hESCs) and iPSCs, can be differentiated into RGCs either via two‐dimensional (2D) culture protocols or by generating 3D retinal organoids.^59^ Proof‐of‐concept studies in animal models show that transplanted human stem cell‐derived RGCs can survive, extend neurites, and even form rudimentary synapses with host retinal circuits.^60^, ^61^ To improve graft survival and ensure appropriate localization within the host ganglion cell layer, recent studies have delivered RGCs on supportive biomaterial scaffolds (e.g., Poly(lactic‐co‐glycolic acid) (PLGA) membranes) or as organized retinal organoid sheets rather than as dissociated single cells. These structured delivery approaches help prevent donor cell dispersion and promote attachment of grafted cells to the inner retinal surface, resulting in improved cell survival and neurite outgrowth in vivo.^60^, ^62^ In a related stem cell–based study, it is demonstrated that human induced retinal ganglion cells (iRGCs), derived from pluripotent stem cells, survived after intravitreal transplantation, migrated into host retinas, and notably conferred neuroprotection to endogenous RGCs following optic nerve injury, indicating both integration potential and therapeutic effect.^63^, ^64^ Moreover, true visual restoration will require that new RGCs not only integrate locally but also project axons through the optic nerve to reinnervate appropriate brain targets. It represents a critical next step in regenerative retinal therapy and a valuable complement to ongoing photoreceptor and RPE replacement strategies.

## 3 ORGANOIDS AND ITS APPLICATION IN RETINAL DISORDERS

### 3.1 The concept and development of organoids

Organoids are in vitro 3D multicellular tissues that recapitulate structural and functional attributes of their in vivo organ counterparts. Derived from induced iPSCs, ESCs, or ASCs, these in vitro models provide a microenvironment wherein cells can interact, differentiate, and organize in a manner that mirrors in vivo organogenesis.^65^ The rise of organoid technology stands poised to revolutionize various domains of biomedical research and has the potential to bridge the chasm between conventional 2D cell cultures and in vivo animal models, both of which have their inherent limitations. The self‐organization of dissociated cells was first observed in sponges.^66^ Starting from the late 20th century, in vivo environments were simulated in cell culture using extracellular matrices (ECMs).^67^ Organoid technologies began to gain significant traction following a landmark study done by Hans Clevers' team, which marks the first time a 3D organoid was established from a single ASC, and where the crypt‐villus structure was cultured in a 3D intestinal organoid in Matrigel without a mesenchymal niche.^68^ Organoid technology offers a transformative approach to understanding human development and disease by accurately simulating the growth and functionality of real organs in vitro. These 3D structures, derived from stem cells, mimic the complex biological processes occurring during organogenesis, making them invaluable for studying developmental stages and disease progression in a controlled environment. As disease models, organoids facilitate in‐depth analysis of pathological processes in diseases like cystic fibrosis or cancer, offering insights that are not easily obtainable through traditional 2D cell cultures.

### 3.2 Retinal organoids

Unlike some evolutionarily primitive vertebrates such as the zebrafish that can regenerate retina cells, the mammalian retina, once damaged in adulthood, poses significant challenges for regeneration, and contemporary treatments for retinal damage are notably restrictive.^69^ Once photoreceptors and RGCs are damaged or lost, they cannot be regenerated, and the inability to regenerate is due to the lack of resident stem cells within the mature human retina that can proliferate and replace lost cells. This limited regenerative ability has driven the field of research into finding alternative solutions, such as stem cell therapy, gene therapy, and the use of retinal organoids. The advent of retinal organoid techniques offers promising avenues for basic researchers to understand the molecular mechanisms of retinal development and pathogenesis, innovate treatment modalities, and explore transplantation possibilities within specific genetic contexts. A myriad of research using model organisms like mice has shed light on retinal development and disease mechanisms. However, differences in cell composition and structure of the human retina compared to these model organisms present translational challenges to clinical applications. Thus, retinal organoids could provide more comprehensive insights.

Prior to the advent of retinal organoid technology, previous studies indicated that when RPCs or other neural stem cells were transplanted into retinas or co‐cultured with retinal cells, they differentiated into retinal cells like photoreceptors, amacrines, or RGCs, but randomly. Later, overexpression of Cone‐Rod Homeobox (Crx) in human retinal stem cells was found to induce differentiation to photoreceptors,^70^ and misexpression of Paired Box 6 (Pax6) led to the generation of retinal ganglion‐like cells.^71^ In 2011, the first generation of retinal organoids was created. Optic vesicle‐like structures were induced from Human Pluripotent Stem Cells (hPSCs). Although early‐stage cell markers for retinal development were shown, along with the presence of photoreceptor‐like cells, the layered structure of the retina was missing.^72^ Consequently, the approach was improved, and optic cups derived from mouse and human ESCs were successfully generated.^73^, ^74^, ^75^

**FIGURE 3:**
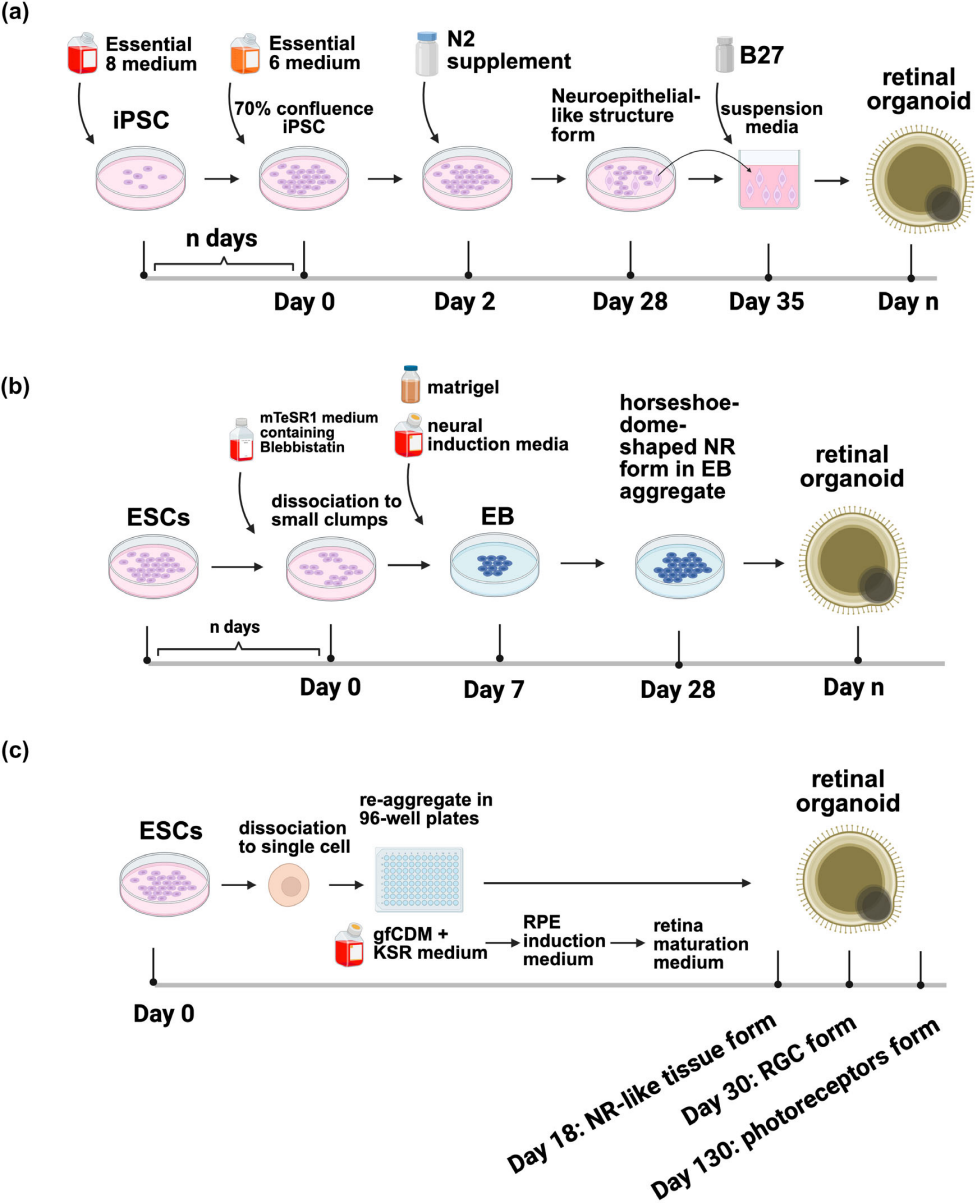
Comparative overview of methodologies for generating retinal organoids from induced pluripotent stem cells/embryonic stem cells (iPSC/ESC). (a) iPSC‐derived retinal organoid formation: iPSCs are cultured to 70% confluence in Essential 8 medium, then switched to Essential 6 medium and supplemented with N2 to induce neuroepithelial‐like structures, which are further matured in suspension media containing B27 to form retinal organoids. (b) Embryonic stem cells (EB)‐mediated ESC‐derived retinal organoid formation: ESCs are dissociated into small clumps and cultured in Maintenance TeSR1 (mTeSR1) medium with Blebbistatin to form EBs, which are then induced to form neuroretina (NR) domains in a neural induction medium before being dissected and matured into retinal organoids. (c) Single cell reaggregation ESC‐derived retinal organoid formation: ESCs are dissociated into single cells and re‐aggregated in Growth factor‐free Chemically Defined Medium (gfCDM) + KnockOut Serum Replacement (KSR) medium. NR‐like tissues are dissected and cultured in retinal pigment epithelium (RPE) induction medium and retina maturation medium, leading to the formation of structured retinal organoids. Created with BioRender.com

Protocols of retinal organoid generation are continuously being refined. Retinal organoid induction techniques can be broadly categorized into three main approaches (Figure 3). The first approach involves transitioning from a 2D to a 3D process and bypassing the embryoid body (EB) phase. After achieving around 70% confluence in iPSC cultures, the medium is switched from Essential 8 to Essential 6 (lacking FGF2 and Transforming Growth Factor Beta (TGFβ)). Around the four‐week mark, self‐organizing neuroepithelial structures emerge. When cultured in B27‐containing suspension media, these organoids sometimes form rosette‐like structures with potential lamination issues, possibly linked to early‐stage N‐[N‐(3,5‐Difluorophenacetyl)‐L‐alanyl]‐S‐phenylglycine t‐butyl ester (a γ‐secretase inhibitor) (DAPT) use, an inhibitor of Notch.^76^ The second method integrates an EB phase. Here, ESCs are dissociated and cultured to form EBs, which then transit into a neural induction medium. By approximately 4 weeks, retina‐like structures appear and are then maintained in suspension for extended culture.^77^ The third and classical technique starts with dissociating ESCs into individual cells and rapidly aggregating them in a specialized medium within non‐adhesive V‐bottomed 96‐well plates. Early Bone Morphogenetic Protein 4 (BMP4) addition, with dilution every few days, enhances neuroepithelium induction efficiency. By Day 18, neuroretina (NR)‐like tissues are excised and cultured further. By Day 30, RGCs appear, and by Day 130, photoreceptors appear.^75^, ^78^ Notably, the latter two methods yield organoids with layers closely mimicking in vivo retinas, which could provide more insight into understanding the development of the retina in vivo.

### 3.3 A comparative analysis of 2D and 3D retinal organoid models

#### 3.3.1 Disease modeling

Disease modeling, a pivotal tool in the realm of medical research, refers to the creation of systems that mimic and predict the progression of a particular disease. By replicating the biological mechanisms and pathology of a disease within a controlled environment, translational researchers can glean insights into disease progression, underlying causes, and potential therapeutic interventions.^79^ In vivo models, typically utilizing animals such as mice or rabbits, replicate the intricate interactions within a whole organism, providing a comprehensive view of a disease's impact on the body. This holistic approach can capture the complexities of organ interactions, immune responses, and metabolic processes. However, ethical concerns, potential differences between animal models and human physiology, and cost can limit their applicability. In contrast, in vitro models, which employ isolated cells or tissues in controlled environments, allow for greater experimental control and specificity. They can yield faster results and can be tailored to study specific cellular or molecular aspects of a disease. Nevertheless, their scope is limited by the inability to replicate the interactions that occur within an entire living organism. Thus, while in vivo models offer a broader, system‐wide perspective, in vitro models provide a focused, controlled environment for detailed investigations.

Traditional 2D cell culture models place cells on artificial matrices, attempting to mimic the natural extracellular matrix, leading to a non‐natural environment and limiting the model's accuracy. Such setups, although efficient, do not offer the biophysical and biochemical cues crucial for cellular activities like 3D models do. Moreover, the monotypic nature of 2D models lacks the complexity of in vivo cell environments. The surface attributes of 2D models significantly influence cell characteristics, including development and differentiation. Current matrices do not replicate dynamic cell‐to‐matrix interactions, making 2D models highly artificial.

**FIGURE 4:**
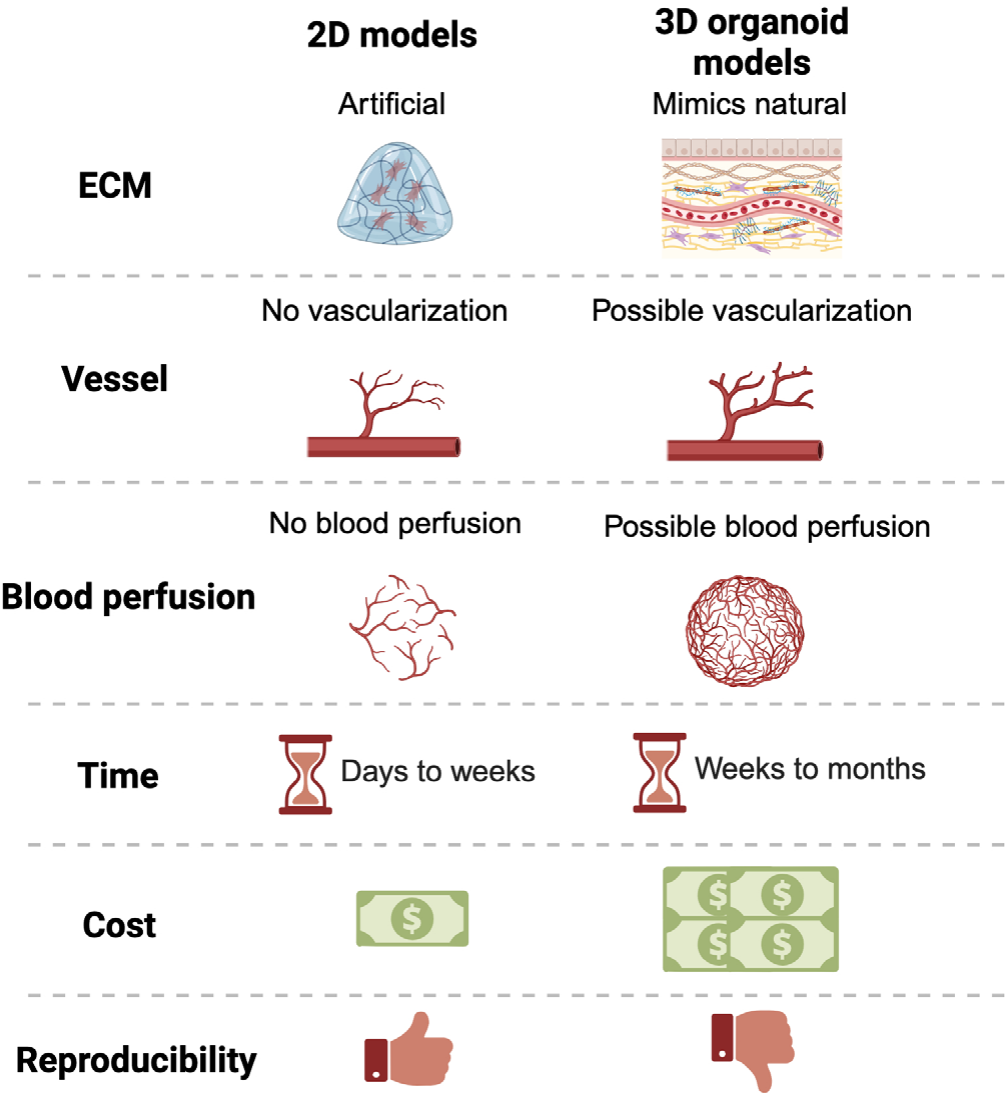
The comparison between two‐dimensional (2D) models and three‐dimensional (3D) organoid models. Compared to 2D models, 3D organoid models mimic natural extracellular matrix (ECM) with better vascularization and blood perfusion. However, they are more time‐consuming, and they cost more for 3D organoid models with low reproducibility. Created with BioRender.com

**FIGURE 5:**
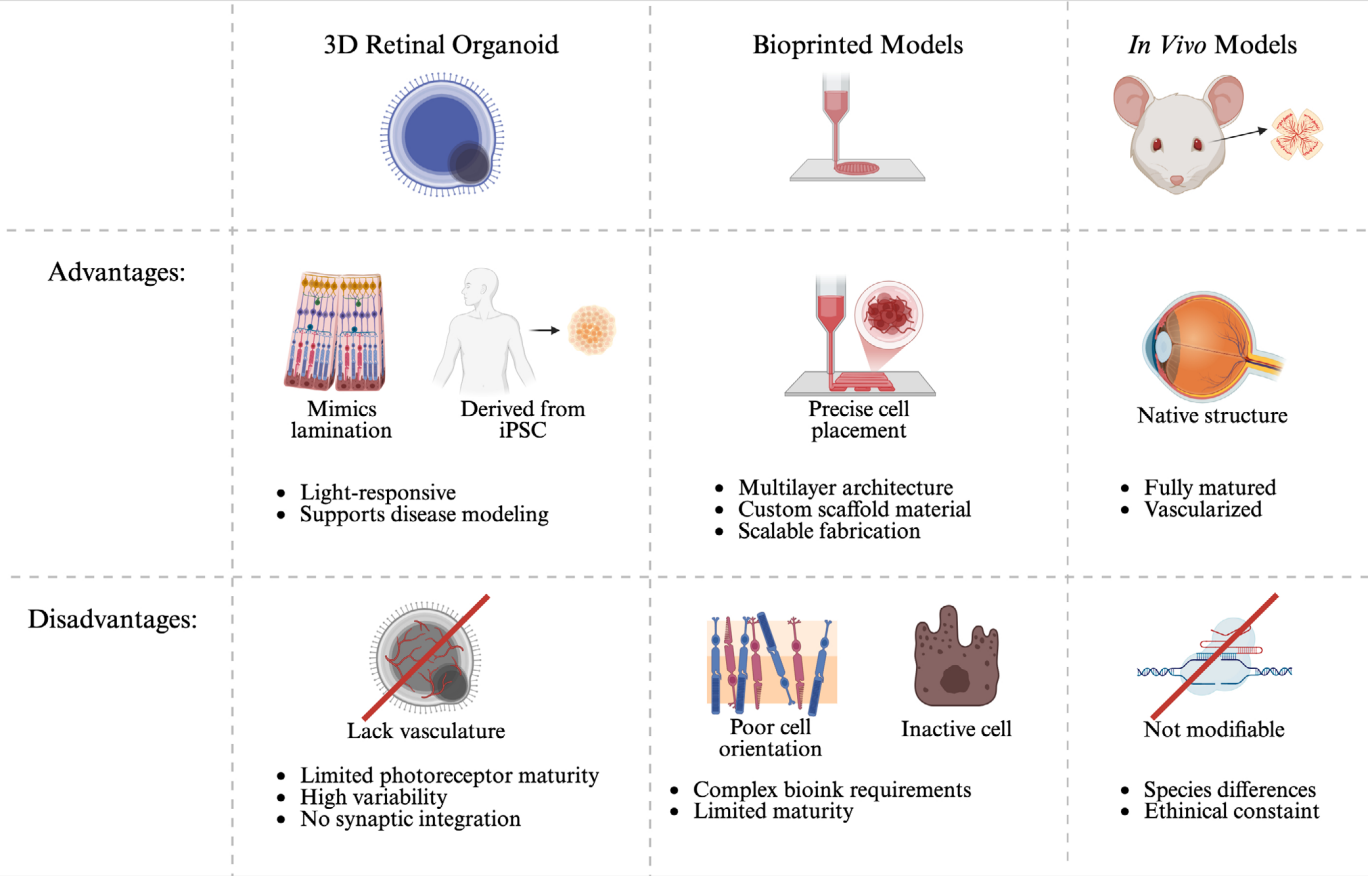
Comparative overview of three‐dimensional (3D) retinal organoids, bioprinted retinal models, and in vivo models. This figure compares 3D retinal organoids, bioprinted models, and in vivo systems in terms of their strengths and limitations for retinal disease modeling and regeneration. Organoids offer lamination and light responsiveness, bioprinted models allow precise cell placement, and in vivo systems provide fully vascularized native structures. Created with BioRender.com. iPSC, induced pluripotent stem cells.

3D organoid models hold many advantages and limitations over traditional 2D models (Figure 4). Apart from being more costly, time‐consuming, and more challenging to set up, 3D organoid models also require vascularization, since it provides insight into drug uptake, metabolism, and circulation of natural organs. While techniques for neovascularization in organoids are still at their infant stage, many studies and attempts have been made. With vascularization, blood perfusion would also be a challenge in creating a 3D organoid, as interactions with immune cells provide insight into the inflammatory responses of the organoid. A 3D organoid model that has both vascularization and blood perfusion would be a powerful tool to study a myriad of inflammatory diseases. Apart from the technical issues of creating a successful organoid, another limitation is its lack of reproducibility. Since cells are self‐organized in organoids, researchers currently have minimal control over the size, shape, cell composition, and molecular characteristics of the organoids. Retinal organoids are often cultured from patient‐derived cells and can serve as a tool to test out novel drugs or therapies before clinical trials. While the autonomous nature of organoids is a remarkable strength, it can also be a challenge in clinical trials where consistent results are required.^80^ This comparison can be further extended to include bioprinted models and in vivo systems mentioned above, offering a more holistic view with their respective strengths and limitations (Figure 5).

#### 3.3.2 Clinical trial

In June 2020, the first human clinical trial employing hiPSC‐retina organoids for patients with advanced RP was approved and initiated at Kobe City Eye Hospital in Japan. This study was supported by the Japan Agency for Medical Research and Development (AMED) and Sumitomo Pharma Co. Ltd. (jRCTa050200027). In the eyes of two patients, three iPSC‐retina sheets were successfully transplanted, demonstrating stable survival for over 1 year in an environment characterized by severe retinal degeneration, all without any serious adverse events. To mitigate any potential risks of rejection, oral cyclosporine was administered for the initial 6 months post‐transplant.^81^

In addition, Lymphocyte Graft Immune Reaction (LGIR) tests, known for their sensitivity in detecting graft rejection, were conducted.^82^ The results confirmed the absence of graft rejection during and after the cessation of cyclosporine administration, validating the stability and safety of the transplantation procedure.^81^

### 3.4 Limitations of retinal organoid technology

Compared to animal models, retinal organoids can faithfully simulate human retinal development, offering greater clinical translational value. However, retinal organoid technology still faces significant limitations that need to be addressed to fully realize its potential.

One major limitation is the heterogeneity observed both across different retinal organoid protocols and among individual organoids. This variability can be attributed to the epigenetic memory of the initial somatic cells. The importance of epigenetic memory was highlighted in the development of retinal cells in 3D cultures. For instance, iPSCs derived from rod photoreceptors retain epigenetic characteristics that enhance their efficiency in forming mature photoreceptors. This finding is crucial for developing therapies for retinal degenerative diseases, as it underscores the potential of using epigenetically primed cells.^83^ To address this, there is a need for developing more standardized protocols that can reduce variability and produce more consistent results. High‐throughput screening techniques could be employed to identify optimal conditions for organoid formation and maturation.

Another significant limitation is the defects in cell maturation and tissue structural integrity within retinal organoids. These organoids often exhibit poorly matured photoreceptors and lack direct contact with the RPE. This lack of structural integrity results in low responsiveness to light stimulation, which is critical for functional retinal studies. Although the photoreceptors in the organoids resemble those in the human retina, discrepancies exist, particularly in the inner retina lamination, which is less similar to the human retina.^84^ This structural limitation impacts the fidelity of retinal organoids in mimicking human retinal functions. Innovative bioengineering approaches, such as co‐culturing retinal organoids with RPE cells or using biocompatible scaffolds, could enhance the structural integrity and functional maturation of these organoids. Additionally, mechanical and biochemical cues that mimic the natural retinal environment could be incorporated into the culture systems to promote better maturation. The low responsiveness of retinal organoids to light stimulation is a significant limitation. To improve this, advanced functional assays that better assess the organoids' physiological responses to light and other stimuli are needed. This could involve the use of optogenetics to directly stimulate and monitor the activity of photoreceptors within the organoids, providing a more accurate assessment of their functionality.

Current retinal organoid protocols also fall short in accurately reflecting the aging effects on progressive neurodegeneration and late‐onset retinal degenerative diseases. Even with prolonged culture periods, the organoids exhibit photoreceptor structures that resemble late fetal stages rather than the more complex and stratified structures of the adult mammalian retina. This developmental gap significantly impacts the organoids' utility in studying late‐onset diseases. Efforts to induce aging‐like features in cells, such as adding stressors to the culture medium, overexpressing aging‐related proteins, and manipulating telomeres, have been experimented within other neurodegenerative disease models. However, these techniques have not yet been successfully adapted for use in 3D retinal tissue models, highlighting a gap in current methodologies.^85^ Addressing this gap is essential to enhance the relevance of retinal organoids for studying and developing treatments for late‐onset retinal diseases. Developing new techniques to simulate the aging process in retinal organoids is crucial. This could involve integrating gene editing technologies, such as CRISPR/Cas9, to introduce aging‐related mutations or using advanced culture systems that can mimic long‐term aging conditions.

Despite these challenges, retinal organoid technology represents a significant advancement in regenerative medicine, particularly for addressing an array of eye diseases. The ability of retinal organoids to more accurately emulate the complex structure and function of the human retina compared to 2D cultures makes them a valuable resource. However, to fully leverage their potential, continued improvements in standardized culture protocols, functional assays, and the incorporation of aged cell models are necessary. These advancements will help enhance the maturity and utility of retinal organoids, paving the way for their use in personalized medicine, transplants, and disease modeling. By overcoming these limitations, retinal organoids could play a pivotal role in the regenerative approaches to various eye diseases, offering new hope for patients with currently untreatable conditions.

Meyer's team has demonstrated robust improvements to existing retinal organoid differentiation protocols that greatly streamline the differentiation process, resulting in highly reproducible retinal organoids relative to their size and shape, and perhaps most importantly, at an efficiency of 100% purity across multiple cell lines. The ability to yield pure populations of retinal organoids with high consistency in their composition across multiple cell lines greatly reduces previous issues with efficiency between cell lines that were difficult to direct to a retinal lineage. The method provides a more standardized and efficient method for generating highly reproducible retinal organoids, greatly facilitating the study of human retinogenesis and providing essential improvements for future disease modeling and pharmacological screening applications.^86^

While retinal organoids hold great promise for personalized medicine and disease modeling, translating these findings into clinical applications remains challenging. Establishing robust preclinical models that can bridge the gap between retinal organoids and human clinical trials is essential. Additionally, regulatory frameworks need to be developed to ensure the safety and efficacy of retinal organoid‐based therapies. For retinal organoid technology to be widely adopted in clinical and research settings, scalability and reproducibility are critical. Efforts should be directed toward developing automated systems for organoid production and analysis, which can streamline the process and reduce manual variability.

## 4 BIOPRINTING APPROACHES

Bioprinting is a method of creating cell patterns and 3D structures using printing techniques. When applied to the retina, bioprinting aims to fabricate retinal tissues for research or potential therapeutic applications. Bioprinting of the retina involves depositing layers of cells, extracellular matrix materials, and sometimes supportive biomaterials in a manner that closely resembles the natural structure and organization of the retina. Bioprinters are specialized devices that deposit cells and biomaterials layer‐by‐layer to construct the desired tissue structure. For the retina, this means creating a multi‐layered tissue that closely mirrors the organization of the natural retina, with its distinct layers and cell types.

There are several different aspects in studying bioprinting of the retina, from cell culture, scaffolds, cell types, and cell orientation, to stiffness. From the very beginning, it is difficult to culture retinal cells in vitro.^87^ Retinal cells need to undergo differentiation for regeneration, but many of those undergo apoptosis, which makes the cell culture difficult.^88^ The structure of the retina is composed of several different layers, which makes it complex in the scaffolds. 3D bioprinting would allow obtaining a precise retina that restores its original morphology and phenotype.^89^ The scaffold can be made in detail while setting the correct parameters, which is also an advantage in 3D bioprinting.^90^

Another advantage of 3D bioprinting is that it can help to incorporate all different cell types into a retina, even though the retina has a high cell diversity. The retina is characterized by this extensive cellular diversity, comprising over 60 distinct cell types.^91^ Arranging different cell layers could be difficult for other manufacturing techniques, but not for 3D bioprinting. Using 3D bioprinting, it is possible to systematically incorporate ganglion cells as the initial layer, followed by bipolar cells in the second layer, cones and rods in the third, and finally, RPE cells in the fourth layer. This precision and layer‐specific approach are what make 3D bioprinting a valuable tool in retinal tissue engineering.^90^

After the cells are stacked into a complex scaffold, it is noted that the cells need to be oriented properly to maintain a function.^92^, ^93^ However, one of the difficult aspects of 3D bioprinting is making the correct orientation. New 3D bioprinting technology is being developed to help achieve more precise control of the overall orientation.^90^

The materials for 3D bioprinting are also important, since the materials and mixtures used need to have a certain level of stiffness that allows the exchange with the damaged retina. It is ideal to make the 3D‐printed retina almost the same as the natural retina.^94^

### 4.1 Microvalve‐based inkjet bioprinting

A structure of the retina was proposed to use microvalve‐based bioprinting. Utilizing microvalve‐based bioprinting, a structure replicating the retina's complexity was successfully fabricated.^95^, ^96^ The primary focus of this paper's 3D bioprinting endeavor was on the generation of the RPE and photoreceptor layers. The RPE layer was composed of ARPE‐19 cells, while the photoreceptor layer was constructed using Y79 cells. The fabrication process commenced with the bioprinting of a first monolayer. This layer was comprised of a mixture of alginate and pluronic, embedded with RPE cells (ARPE‐19), and was laid down onto a preformed membrane. Following this, the second layer was applied. This layer, sharing the same material composition, was enriched with photoreceptor cells (Y79), forming a crucial part of the retinal structure. The viability remained intact, and there was a progressive increase in cell density over time. This initial demonstration successfully showed that it is possible to create a structure with properties akin to the retina while maintaining robust cell viability and compatibility. The results of adequate cell viability and morphology, as well as the quality of bioprinted cells, stand for their potential in broad applications in retina‐related research, not only in disease models but also in potential drug testing and treatment options.^95^ After printing, the fabricated tissue often requires a period of maturation in a controlled environment. This allows cells to further differentiate, form connections, and develop tissue‐specific functions.^90^

### 4.2 Laser‐assisted 3D bioprinting

Another method of 3D bioprinting was developed by culturing in vitro photoreceptor cells.^97^ Photoreceptor cells that are cultured in isolation in vitro without the presence of the extracellular matrix (ECM) undergo a significant number of morphological changes and apoptosis. In the human retina, the primary composition of the ECM is hyaluronic acid (HA), a negatively charged polysaccharide. To combat this challenge, hydrogel is derived from hyaluronic acid modified with glycidyl methacrylate (HA‐GM), which serves as the primary bioink. To construct a layer‐by‐layer structure, a rapid 3D printing technique is employed. This method utilizes a light source of 365 nm wavelength to initiate photo‐polymerization, solidifying the hydrogel‐cell mixture layer by layer. Designs for the intended structures are created using software like Solid Works®, which are then converted into digital mask patterns. These patterns are subsequently transferred to a digital mirror array device (DMD) chip that acts as an optical mask. When Ultraviolet (UV) light passes through this mask and its associated lenses, it projects precise patterns onto the hydrogel and cell solution. Wherever the light strikes, polymerization occurs, forming the desired structure. The stage holding the solution is computer‐guided and moves in all three dimensions, facilitating the creation of multi‐layered structures. The first layer consists of a mixture of RPEs purchased from American Type Culture Collection (ATCC) and the hydrogel. The second layer consists of a mixture of human fetal retinal progenitor cells (fRPCs) harvested from 20‐ to 24‐week‐old fetuses and the hydrogel. After one layer is printed, it is rinsed, and the next layer's solution is introduced, continuing the process until the entire structure is complete. The viability of the hydrogel and cell combination can be up to 70%.^97^

### 4.3 Thermal inkjet 3D bioprinting combined with electrospinning

Thermal inkjet was used to combine electrospinning to conduct 3D bioprinting to print in vitro RGCs. In the study, RGCs were derived from Sprague Dawley rats and processed to create a single cell suspension. Radial scaffolds were produced using electrospinning, wherein polylactic acid was dissolved, pumped through a needle, and charged to create fine fibers. For the actual bioprinting, an HP TIPS thermal inkjet printing system was employed. This system allows fine control over the droplet dispensing process with interchangeable print head nozzles of 80 μm diameter. The printing parameters, refined through pilot tests, had a dispensing frequency of 50 Hz and a voltage of 27 V. For the placement of cells, an XY actuator system was used, synchronized with the print head to dispense 10 droplets per 1 mm of motion. Recognizing the need to stabilize cell positions post‐printing, the media was mixed with alginate to increase its viscosity. Two printing patterns were utilized: parallel lines and a detailed retinal pattern featuring a radially spoked circle. This innovative bioprinting method offers promise for precise RGC placement and retinal tissue engineering.^98^

Overall, 3D bioprinting is a highly promising technology with its ability to create complex, functional tissues using living cells. Its proven versatility, demonstrated successes, and continuous advancements underscore its significance. Micro‐valved‐based inkjet printing offers precision and versatility with low shear stress on cells but faces challenges with complexity and valve maintenance. Laser‐assisted bioprinting excels in high‐resolution printing and avoids nozzle clogging yet requires costly equipment and is limited by material compatibility and technical expertise. A thermal inkjet combined with electrospinning provides fiber reinforcement and scalability, suitable for diverse materials. However, it may compromise cell viability due to heat sensitivity and has limitations in resolution and process complexity (Table 3).

**TABLE 3:** Three‐dimensional (3D) bioprinting techniques.

3D bioprinting technique	Cells used	Bioink for ECM	Preclinical directions
Microvalve‐based inkjet	hRPE cell line (ARPE‐19), human retinoblastoma cell line (Y79)	DMEM:F12	Bilayer retinal tissue with gradient cell density for in vitro modeling^90^, ^96^
Laser‐assisted	RPE and fRPCs	HA‐GM and PEG‐RGDS	Precise photoreceptor placement using laser‐assisted bioprinting^97^, ^99^
Thermal inkjet combined with electro spinning	RGCs	Alginate and culture medium	Thermal inkjet printed scaffolds for RGC alignment and viability^98^, ^100^

Abbreviations: DMEM, Dulbecco’s Modified Eagle Medium; ECM, extracellular matrix; fRPCs, fetal retinal progenitor cells; HA‐GM, hyaluronic acid modified with glycidyl methacrylate; hRPE, Human Retinal Pigment Epithelium (cells); PEG, Polyethylene Glycol; RGC, retinal ganglion cell; RGDS, Arginine‐Glycine‐Aspartic Acid‐Serine peptide; RPE, retinal pigment epithelium.

### 4.4 Other Relevant 3D Technologies

While bioprinting is a leading method in retina tissue engineering, there are other 3D techniques, including electrospinning, macro and nanoelectromechanical systems (MEMS/NEMS), and porous gel scaffolds, that also support the field of cell transplantation. Electrospinning fabricates nanoscale fibrous scaffolds that closely mimic the native extracellular matrix, which supports cellular adhesion and differentiation. This allows them to align with RPE and photoreceptor support. For instance, poly(ε‐caprolactone) (PCL) electrospun scaffolds incorporating human amniotic membrane powder have demonstrated improved hydrophilicity and proliferation of ARPE‐19 cells, a type of immortalized human RPE cell.^101^ Radially aligned electrospun scaffolds integrated with 3D‐printed features have also guided RGC positioning and neurite extension.^98^ A recent study showed that PCL–laminin or PCL–chitosan fibers improved RPC attachment and photoreceptor marker expression. However, electrospun scaffolds often suffer from batch‐to‐batch variability, limited cell penetration, and diffusion due to their dense structure, and degradation and immunogenicity remain concerns.^102^

MEMS and NEMS technologies offer precise microscale control for delivering mechanical or electrical stimuli. MEMS platforms have been used to stimulate RGCs, aiding in the modeling of retinal circuitry and the assessment of functional integration. MEMS‐based electrode arrays have been developed for subretinal and epiretinal stimulation to restore vision by interfacing with retinal neurons.^103^ The MEMS‐EYE project has also introduced the concept of large‐scale integrated arrays of MEMS and NEMS that process images without conventional electronics, utilizing the physical properties of coupled resonators for the recognition of neuromorphic patterns.^104^ However, scalability, specialized fabrication, and material biocompatibility remain limiting factors.

Porous gel‐based systems create hydrated environments that mimic the ECM and allow minimally invasive delivery. Hydrogels have made significant progress in AMD, as they can serve as effective scaffolds for RPE cell transplantation by promoting cell survival and integration,^105^ and for drug delivery by enabling sustained therapeutic release.^106^ Co‐culture hydrogel platforms are also being explored for retinal tissue reconstruction.^107^ However, balancing pore size, stiffness, and gelation kinetics is necessary to ensure mechanical stability during implantation.^108^

Overall, while these approaches show promising potential for retinal cell regeneration, they each present certain limitations, which makes 3D bioprinting the most optimal choice for now.

## 5 CONCLUSION

While holding tremendous promises, retinal organoids confront several significant challenges that impede their application in drug development and therapeutic interventions (Table 4). A primary issue is the labor‐intensive and time‐consuming nature of the culture process. This not only limits scalability for high‐throughput screenings but also introduces inefficiencies that are not sustainable in large‐scale operations. During extended culture periods, there is gradual degeneration of the inner cell layers, particularly the ganglion cell layer. The lack of an intrinsic vascular system within the organoids contributes to a hypoxic environment, leading to malnutrition of inner cell populations. Additionally, the lack of synaptic connections with the optic center further exacerbates this degeneration, compromising the assessment of functional retinal circuitry in these models.^110^ Heterogeneity and variability among retinal organoids also pose a significant challenge for their reproducibility.^109^ The variability manifests not just across different cell sources but also within the same batch of derived organoids. This heterogeneity affects not only the induction efficiency but also the resulting cell composition, making it difficult to establish a stable retinal organoid model. The lack of functional vascularization and innervation remains a hurdle for both organoid and bioprinting approaches for the retina. Developing and selecting suitable biologically functional bioinks, combined with correct cell sourcing, bioprinting remains very challenging in the sense of creating reproducible and consistent results.^90^ These challenges collectively represent a substantial barrier to the reliable and efficient use of 3D technology for the retina as models for retinal diseases and platforms for drug discovery and development. The future direction lies in overcoming the current limitations to fully harness their potential for disease modeling, drug discovery, and regenerative therapies. Advanced techniques such as microfluidics, which can provide a more physiological environment for organoid development, may offer solutions to the problem of inner cell layer degeneration by simulating vascularization and facilitating proper nutrient and oxygen supplies.^69^ Compared to traditional 2D cell culture models, which are non‐natural and accuracy‐limited, 3D models are more delicate, especially in their capability for neovascularization and blood perfusion, which better mimics the extracellular matrix. However, 3D models are relatively more costly, time‐consuming, and challenging to set up. To complement retinal organoids, bioprinting and scaffold‐based platforms offer additional 3D approaches that may accelerate integration and improve reproducibility. Electrospinning technologies and MEMS‐based constructs could support layered retinal cell architectures with embedded guidance cues and better mechanical stability. These systems should be further optimized for retinal‐specific applications. Future developments in automation and robotics could greatly resolve present challenges and allow the implementation in a clinical setting of retinal organoids in disease modeling and regeneration to be more pragmatic. Future research on retinal organoid technology is poised at the brink of transforming theoretical models into practical clinical solutions. The recent breakthroughs—demonstrating the organoids' ability to mimic human retinal responses to light and to form connections between cells—underscore their potential for repairing retinal damage and restoring vision. Key to transitioning these advancements into the clinic will be rigorous clinical trials aimed at establishing the safety, efficacy, and optimal application methods for organoid‐derived therapies.

**TABLE 4:** Retinal organoid applications and limitations in preclinical studies.

Preclinical applications	Advantages	Limitations	Available clinical trials
Disease modeling (RP, AMD, glaucoma)^81^, ^84^	Mimics human development and pathology	Immature photoreceptors	NCT03853252
Cell transplantation^19^, ^75^	Tests survival and integration in retina	Structural integration issues, variability among organoids	NCT04604899 (in treating RP)
Light responsiveness and function^83^	Enables optical stimulation studies, early proof‐of‐concept for vision restoration	Limited lamination and synaptic connectivity	N/A
Drug screening and toxicity^109^	Human‐relevant drug response	Low reproducibility	N/A

Abbreviations: AMD, age‐related macular degeneration; RP, retinitis pigmentosa.

Currently, there are still research gaps in 3D retinal regeneration, regarding the integration and functionality after the transplant of stem cells or organoids. Also, more clinical trials have to be done in long‐term viability and efficacy studies in the human body. The use of ESCs and iPSCs would also need thorough ethical and regulatory considerations.

## 6 FUTURE DIRECTIONS AND PERSPECTIVES

Emerging 3D retinal platforms are addressed to transform both basic science and translational ophthalmology. Unlike conventional 2D cultures, 3D retinal organoids and bioprinted tissues recapitulate the eye's layered architecture and cellular diversity. For example, human stem cell–derived retinal organoids closely mimic in vivo development and contain all major retinal cell types in correct organization. This biomimicry enables new insights into normal development and disease mechanisms. 3D models capture cell–cell and cell–matrix interactions that 2D systems do not, allowing researchers to study retinal circuitry and pathology with unprecedented fidelity. Retinal organoids have already been used for high‐throughput drug screening and disease modeling that better predict human responses. Integrating organoids with microfluidic “retina‐on‐chip” devices or patient‐derived iPSC lines can incorporate genetic and individual variability, advancing personalized medicine. As noted, coupling organoids with engineering technologies (e.g. microfluidics, bioprinting) has achieved “unparalleled biomimicry” of the human retina, which accelerates drug discovery and translational research. In the future, building biobanks of diverse retinal organoids will allow screening therapies in defined genetic contexts, directly linking laboratory models to patient care^111^, ^112^

3D bioprinting offers a complementary path toward regenerative therapies. Recent advances in bioinks and fabrication methods enable layered retinal constructs that recreate key structural and biochemical cues. Though challenges remain, such as ensuring long‐term cell viability, vascularization, and immune integration, the progress is steady. A recent study emphasized that refining biomaterials and scaffolds will be essential to generate fully functional, transplantable retinal grafts.^113^ Overall, sustained interdisciplinary efforts are expected to yield bioprinted retinal tissues suitable for drug testing and eventually for vision‐restoring grafts.

In summary, the integration of 3D organoids, bioprinted models, and related platforms is reshaping retinal research and therapy. By capturing patient‐specific biology and complex cellular interactions, these tools will enhance disease modeling, enable personalized drug screening, and help translate discoveries from bench to bedside.^111^, ^112^ The next decade will likely see these technologies mature into routine methods for studying retinal development, degeneration, and for testing innovative treatments.

## AUTHOR CONTRIBUTIONS

**YW**: Writing—original draft, writing—review and editing. **DJ**: Writing—original draft, writing—review and editing. **QW**: Writing—review and editing. **HG**: Writing—review and editing. **YC**: Writing—review and editing. **YL**: Writing—review and editing. **FT**: Writing—review and editing.

## FUNDING INFORMATION

The author(s) declare financial support was received for the research, authorship, and/or publication of this article. Dr. Feng Tian is financially supported by NIH/NEI K99/R00 grant (K99EY032181).

## CONFLICT OF INTEREST STATEMENT

All financial, commercial, or other relationships that might be perceived by the academic community as representing a potential conflict of interest must be disclosed. If no such relationship exists, authors will be asked to confirm the following statement: FT and DJ are co‐founders of Regenerative AI. The other authors declare no competing interests.

## Data Availability

This review does not report new data. All data discussed are available in the cited literature. This review does not involve any studies with human participants or animals performed by any of the authors.
